# Ocean acidification during prefertilization chemical communication affects sperm success

**DOI:** 10.1002/ece3.5720

**Published:** 2019-10-16

**Authors:** Rowan A. Lymbery, W. Jason Kennington, Christopher E. Cornwall, Jonathan P. Evans

**Affiliations:** ^1^ Centre for Evolutionary Biology School of Biological Sciences University of Western Australia Crawley WA Australia; ^2^ School of Biological Sciences Victoria University of Wellington Wellington New Zealand

**Keywords:** broadcast spawning, climate change, egg chemoattractants, ocean acidification, sperm chemotaxis, sperm‐egg interaction

## Abstract

Ocean acidification (OA) poses a major threat to marine organisms, particularly during reproduction when externally shed gametes are vulnerable to changes in seawater pH. Accordingly, several studies on OA have focused on how changes in seawater pH influence sperm behavior and/or rates of in vitro fertilization. By contrast, few studies have examined how pH influences prefertilization gamete interactions, which are crucial during natural spawning events in most externally fertilizing taxa. One mechanism of gamete interaction that forms an important component of fertilization in most taxa is communication between sperm and egg‐derived chemicals. These chemical signals, along with the physiological responses in sperm they elicit, are likely to be highly sensitive to changes in seawater chemistry. In this study, we experimentally tested this possibility using the blue mussel, *Mytilus galloprovincialis*, a species in which females have been shown to use egg‐derived chemicals to promote the success of sperm from genetically compatible males. We conducted trials in which sperm were allowed to swim in gradients of egg‐derived chemicals under different seawater CO_2_ (and therefore pH) treatments. We found that sperm had elevated fertilization rates after swimming in the presence of egg‐derived chemicals in low pH (pH 7.6) compared with ambient (pH 8.0) seawater. This observed effect could have important implications for the reproductive fitness of external fertilizers, where gamete compatibility plays a critical role in modulating reproduction in many species. For example, elevated sperm fertilization rates might disrupt the eggs' capacity to avoid fertilizations by genetically incompatible sperm. Our findings highlight the need to understand how OA affects the multiple stages of sperm‐egg interactions and to develop approaches that disentangle the implications of OA for female, male, and population fitness.

## Graphical abstract

Gametes of externally fertilizing marine organisms are expected to be highly sensitive to ocean acidification (OA), and yet we know little about affects of OA on specific sperm‐egg interactions. Here, we show that OA affects signalling between sperm and eggs via egg‐derived chemicals, with subsequent changes in fertilization rates.

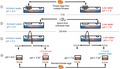

## 1 INTRODUCTION

The current rise in atmospheric carbon dioxide (CO_2_) due to anthropogenic emissions is leading to rapid climate change and unprecedented levels of environmental disturbance. Many of these effects manifest in the world's oceans, where seawater not only acts as a sink for excess global heat but also as a store for human‐produced CO_2_; over the industrial period, the oceans have absorbed approximately 30% of the gas produced anthropogenically per year (Rhein et al., 2013; Sabine et al., 2004). This increase in CO_2_ is altering the carbonate chemistry of seawater and consequently reducing its pH, a process known as ocean acidification (OA) (Caldeira & Wickett, 2005). OA is now recognized as a major threat to marine organisms and a substantial literature has documented impacts of OA on the early developmental stages of species that form calcium carbonate shells, due to reduced carbonate availability or increased hydrogen ions in seawater (reviewed in Byrne, 2011; Doney, Fabry, Feely, & Kleypas, 2009; Kroeker et al., 2013). However, OA also has the potential to impact many species at much earlier life‐history stages during reproduction, for example, by affecting gametes and fertilization (Byrne, 2011). Since the majority of marine species are external fertilizers, the reproductive capacity of most ocean species is likely to be particularly impacted by changes in oceanic chemistry.

Relatively few studies have examined early reproductive stages of external fertilizers under OA, and among those that have, most have focused on in vitro fertilization assays, or on sperm swimming behavior (due to the well‐documented importance of intracellular pH for sperm function; Nishigaki et al., 2014). Results from these investigations have so far been mixed; some studies have reported negative effects of OA on sperm motility (e.g., Campbell, Levitan, Hosken, & Lewis, 2016; Morita et al., 2010; Nakamura & Morita, 2012; Schlegel, Havenhand, Gillings, & Williamson, 2012; Vihtakari et al., 2013) while others have revealed negligible or even positive effects (Caldwell et al., 2011; Eads, Kennington, & Evans, 2016; Graham et al., 2016; Havenhand & Schlegel, 2009). Furthermore, there are inconsistent associations between OA‐induced changes in sperm motility and in vitro fertilization rates (reviewed in Byrne, 2011; Ross, Parker, O'Connor, & Bailey, 2011), and thus, the fitness consequences of the effects of OA on gametes remain elusive.

In external fertilizers, fertilization is determined by complex, multifaceted interactions among gametes (e.g., see Figure 1 in Evans & Sherman, 2013). These include initial gamete dispersal, long‐distance gamete communication and attraction through egg‐derived chemicals, sperm capacitation, and sperm‐egg fusion mediated by surface interactions (Beekman, Nieuwenhuis, Ortiz‐Barrientos, & Evans, 2016; Kekäläinen & Evans, 2018). Therefore, simple assays of sperm behavior in isolation (i.e., in the absence of eggs or female secretions) or in vitro fertilization (where sperm are mixed directly with eggs) may be poor predictors of realistic reproductive success (Lüpold & Pitnick, 2018). Therefore, to elucidate the impacts of environmental changes such as OA on reproduction, we require mechanistic approaches that disentangle the different stages of the external fertilization process. Intriguing recent evidence suggests that prefertilization gamete interactions might be sensitive to seawater pH changes; for example, in sea urchins, low pH alters the motility of sperm in egg chemical solutions and reduces the size of the egg jelly layer (which contains sperm‐attracting chemicals) (Foo Byrne & Cristina, 2018; Foo, Deaker, & Byrne, 2018). However, it has yet to be determined how such effects link to reproductive (i.e., fertilization) outcomes.

**Figure 1:**
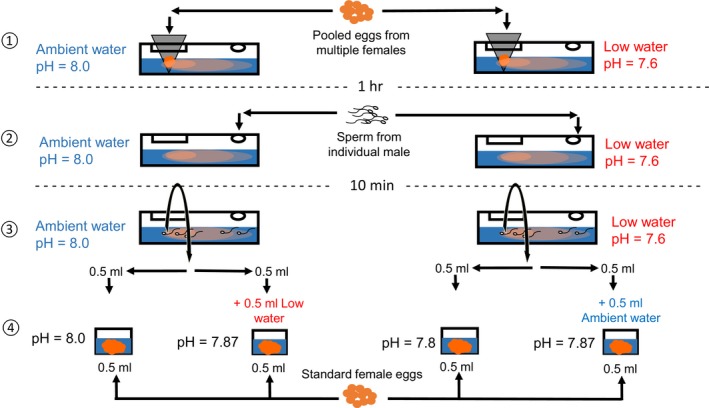
Overview of experimental design showing chemotaxis and fertilization procedures for sperm from an individual male in one block. Step 1: Two chemotaxis chambers were prepared, one with ambient pH and one with low pH seawater, and aliquots of pooled eggs placed in filter mesh at one end of each chamber. Step 2: After 1 hr, eggs were removed and aliquots of sperm from the male placed in the opposite ends of the chambers. Step 3: After 10 min, aliquots were taken from the center of the chemoattractant gradient for each chamber, split into two and mixed with separate aliquots of washed eggs from the standard female. Step 4: For one fertilization mix per chemotaxis trial, no further seawater was added (unstandardized fertilization pH). For the other fertilization mix in each trial, an aliquot of seawater from the opposite treatment was added (low pH water for ambient chemotaxis trial, and ambient pH water for low chemotaxis trial; standardized fertilization pH). These procedures were repeated for every male in each block (*n* = 26 males total in 6 blocks)

In this study, we explore the effects of OA during prefertilization sperm‐egg interactions in the mussel *Mytilus galloprovincialis*. This species has emerged as a model system for the study of such prefertilization processes. For example, a series of recent studies on *M. galloprovincialis* has revealed complex effects of egg‐derived chemicals (ECs) in the seawater on subsequent sperm success. These studies demonstrated that, among intraspecific male–female pairings, ECs differentially moderate patterns of sperm attraction (Evans, García‐González, Almbro, Robinson, & Fitzpatrick, 2012), swimming behavior (Oliver & Evans, 2014), and changes to sperm surface physiology (e.g., acrosome reaction and arrangement of glycan molecules; Kekäläinen & Evans, 2016). These effects of ECs on sperm behavior and physiology are likely to explain how females regulate fertilization in favor of genetically compatible males when ejaculates from different males compete (Lymbery, Kennington, & Evans, 2017). However, whether OA alters these prefertilization processes has not yet been tested. A recent study on *M. galloprovincialis* reported evidence that OA has slight negative effects on sperm motility in seawater and in vitro fertilization rates (Eads et al., 2016), although it is unclear whether these patterns reflect biologically realistic sperm‐egg interactions.

Here, we determine whether changes in ocean pH, as predicted for near‐future ocean acidification by the Intergovernmental Panel on Climate Change (IPCC, 2013), have implications for sperm‐egg interaction and fertilization. We employ an experimental design that combines different seawater CO_2_ (and therefore pH) treatments with multistep experimental trials that separate sperm swimming in a gradient of ECs from fertilization. These experimental procedures enable us to (a) isolate the effect of seawater pH on sperm in a realistic environment of ECs prior to fertilization and (b) measure the outcomes of any observed effect in terms of overall fertilization rates. As such, our study provides much‐needed mechanistic insight into the way that changes in seawater chemistry influence sperm‐female interactions under predicted levels of OA.

## 2 MATERIALS AND METHODS

### 2.1 Study species and spawning

*Mytilus galloprovincialis* is a sessile, marine bivalve mollusk that forms large intertidal aggregations in temperate regions (Daguin & Borsa, 2000), including the southern Australian coastline (Westfall & Gardner, 2010). *Mytilus galloprovincialis* is a gonochoristic (sexes separated into physically distinct individuals) broadcast spawner, undergoing a series of synchronized spawning events during the reproductive season (June‐September in Western Australia). We collected adult mussels from Woodman Point, Western Australia (32°14′03.6″S, 115°76′25″E) during the 2018 spawning season. Mussels were induced to spawn in the laboratory on the day of collection, using a temperature increase from ambient (approximately 21–22°C in the laboratory) to 28°C (Lymbery, Kennington, & Evans, 2016). As soon as an individual began spawning and its sex was determined, it was washed to remove any contaminating gametes and placed in an individual 250 ml plastic cup with enough filtered seawater (FSW; see below) to cover it. When gametes were suitably dense (within 30 min of spawning), we removed the mussels and estimated gamete concentrations. Egg concentrations were estimated by counting the number of cells in a homogenized 5 µl subsample, and sperm concentrations were estimated in subsamples fixed with 1% formalin using an improved Neubauer hemocytometer (Hirschmann Laborgeräte). We used these estimates to adjust gametes to the concentrations required for experimental trials (see below).

### 2.2 Seawater treatments and carbonate chemistry

We conducted our experiments over a series of days for practical purposes, where each experimental day constituted a “block” (*n* = 6 blocks in total) using different groups of animals and new batches of seawater. In this way, seawater treatments were replicated as recommended by Cornwall and Hurd (2016). We prepared experimental seawater synthetically by dissolving Ocean Nature Sea Salt (Aquasonic) in deionized water to a salinity of 35 psu. This water was then run through a series of mechanical filters (final mesh size = 5 µm), a carbon filter, and treated to ultraviolet sterilisation, to remove any contaminants that could affect carbonate chemistry parameters (filtered seawater is hereafter referred to as FSW). For each block, we prepared 10 L batches of FSW for each of two pH treatments: “ambient” (pH ~ 8.0), a treatment reflecting current sea surface conditions, and”low” (pH ~ 7.6), an experimentally adjusted pH designed to simulate predicted end‐of‐century conditions under a high CO_2_ emissions scenario (representative concentration pathway 8.5; IPCC, 2013). The pH of the “low” treatment was experimentally adjusted by bubbling pure, commercial‐grade CO_2_ through the FSW, with the pH change monitored using a Blueline 24 pH electrode attached to a HandyLab 100 meter (Xylem Analytics).

Following the experimental manipulation of FSW in each block, pH on the total scale (pH_T_) was measured potentiometrically in the FSW batches by calibrating the electrode against a Tris buffer (Dickson, Sabine, & Christian, 2007). Tris calibrations were conducted at four temperatures covering the range observed in the FSW. Across different experimental days (blocks), the ambient laboratory temperature varied slightly (mean ± standard error of FSW temperature = 21.77 ± 0.34°C); however, within each experimental block the FSW temperature was constant for the duration of the chemoattraction trials (see below for trial details) and did not differ between treatment batches. Therefore, any temperature effects on sperm behavior are incorporated into the among‐block variance, (see Data analyses) and do not confound pH treatments.

Subsamples of each FSW batch were used to measure total alkalinity (TA) via potentiometric titration (Dickson et al., 2007) in a T50 Titrator (Mettler‐Toledo). Titration of certified reference material (CRM; batch 174; Scripps Institute of Oceanography, UCSD) returned TA within 6 µmol/kg of the certified value. Partial pressure of CO_2_ (Pco_2_) and total dissolved inorganic carbon (DIC) in each FSW batch were calculated from pH_T_, TA, temperature, and salinity data using the “seacarb” package (Gattuso, Epitalon, Lavigne, & Orr, 2018) in R version 3.5.1 (R Core Team, 2019).

### 2.3 Experimental design: sperm‐EC interaction and fertilization trials

Sperm were collected from 2 to 5 individual males per block (*n* = 26 males in total across six blocks) and standardized to 5 × 10^6^ cells/ml in ambient FSW. The sperm‐EC interaction trials were conducted using the chambers described in Lymbery et al. (2017). Two of these chambers were prepared per focal male (Figure 1; *n* = 52 trials in total across the experiment); one was filled with 5 ml of FSW from the ambient pH treatment, and the other filled with 5 ml from the low pH treatment. Eggs were collected from 3–5 females per block, standardized to 5 × 10^4^ cells/ml in ambient FSW and then mixed in equal volumes to form a common egg pool. Pooling eggs from multiple females increases the probability that sperm will respond strongly to some of the ECs in the chambers. Specifically, sperm responses to ECs in *M. galloprovincialis* are characterized by variation in male–female compatibility (Evans et al., 2012; Oliver & Evans, 2014); if individual females were used, then by chance sperm from some males would have weak responses to ECs and our power to detect changes in sperm‐egg communication across treatments would be low. Aliquots of the egg pool (2 ml) were added to filter mesh sacks (pore size = 30 µm; small enough to retain eggs while allowing ECs to leach out) at one end of each chamber (Figure 1, step 1). The eggs were left in the filter sacks for 1 hr to establish an EC gradient (as per Lymbery et al., 2017). The filter sacks and eggs were then removed, and 1 ml aliquots of sperm from the focal male added to each chamber (ambient and low pH chambers), at the opposite end from where the eggs were removed (Figure 1, step 2). Sperm swam in the chambers for 10 min, then subsamples were taken from the source of the EC gradient, that is where the eggs had previously been (Figure 1, step 3; a single 1 ml subsample was taken from each chamber, then split into two 0.5 ml aliquots for the different fertilization designs; see below). We have previously employed a similar experimental setup to show that sperm orient toward the source of ECs (Evans et al., 2012) and that these experimental chambers can be used to measure differential responses to ECs among sperm from rival males (Lymbery et al., 2017).

Following the methods in Lymbery et al. (2017), we estimated effects of ECs on sperm by using the subsamples taken from the chambers in subsequent fertilization trials with eggs from a separate female (i.e., a different individual to those used for the EC pool; hereafter referred to as a “standard” female, as the same egg donor was used for all trials within a block). There are two reasons for using this procedure rather than simply counting the number of sperm in each subsample. First, these trials allow us to draw a direct link between differential prefertilization effects on sperm and reproductive outcomes (fertilization success). Second, the subsamples of sperm taken from the chamber at step 3 of Figure 1 are at relatively low concentrations, containing only a subset of cells that had successfully reached the center of the gradient. While there are sufficient sperm in the entire subsamples to produce variation in fertilization rates in subsequent trials (Lymbery et al., 2017), it would be impractical to attempt to count sperm from these samples with a hemocytometer (sperm concentrations would be too low for the very small volumes required by hemocytometers).

The same standard female was used for all trials within a single block. While there may be differences in sperm‐egg surface compatibility between different males and standard females, our paired design means these would be incorporated in overall male variation and would not confound pH treatment effects. These eggs were washed clean of their own ECs immediately prior to fertilization trials by rinsing with FSW through 30 µm filter mesh, then standardized to 5 × 10^4^ cells/ml (in ambient FSW). Separate 0.5 ml aliquots of standard female eggs were prepared for each treatment per male, and 0.5 ml of the sperm subsample from each chamber (ambient or low pH) was added to the separate egg aliquots; that is for each treatment‐by‐male combination in the chambers, aliquots were added to two separate fertilization trials (Figure 1, step 4).

Addition of sperm subsamples from the chambers would have altered the pH of the fertilization mix, meaning that any “treatment” effects detected from fertilization data could have been due to either (a) differential effects on sperm in the chambers, or (b) differential sperm‐egg fusion and zygote development. To separate these possibilities, we performed two fertilization trials for each pH treatment (Figure 1, step 4; *n* = 104 fertilization trials in total, 2 per chemotaxis trial and 4 per male). In “unstandardized” fertilizations, sperm and eggs were mixed as described above, meaning pH would have differed across treatments in both the sperm‐EC chambers and the fertilization mix. In “standardized” fertilizations, sperm from the chambers were added to eggs, along with a 0.5 ml aliquot of the opposite FSW treatment (i.e., ambient FSW for treated sperm and treated FSW for ambient sperm), meaning pH would differ between treatments in the sperm‐EC chambers, but not in the fertilization mixes. Therefore, if a treatment effect was consistent across both fertilization designs, the effect could be attributed to the pH of the sperm‐EC chambers. If there was evidence that the pH of the fertilization mixes was influencing the treatment effect (i.e., a treatment‐by‐design interaction), it could be complex to interpret, given the different pH histories experience by sperm and standard female eggs. However, the effect of seawater pH directly on sperm‐egg fusion and fertilization is not within the scope of our study; the purpose of the fertilization trials here is to determine the flow‐on effects of prefertilization interactions for reproductive success. Therefore, the only aim of comparing the different fertilization designs is to determine whether a pH effect can be isolated to the prefertilization sperm‐EC stage.

Our experimental protocols meant that the standardized fertilization mixes would have had higher volumes, and therefore lower gamete densities, than the unstandardized mixes, which might lead to slight differences in fertilization rates. However, within each fertilization design (standardized or unstandardized) the volume was equivalent for the two treatments (sperm from ambient or low pH). We can therefore be confident that any pH treatment effects would not be confounded by fertilization volume. All fertilizations were allowed to proceed for 2 hr and then fixed in 1% buffered formalin until required for the assessment of fertilization rates. To measure fertilization rates, we assayed a haphazard sample of 100 eggs and scored the proportion undergoing polar body formation and/or cell division.

### 2.4 Data analyses

Analyses were conducted using R version 3.5.1 (R Core Team, 2019). The proportion (out of 100) of fertilized eggs in each sample was analyzed as a binomial response variable (i.e., as the number of successful fertilizations out of 100 eggs) using a generalized linear mixed‐effects model (GLMM) with a logit link function in the “lme4” package (Bates, Macechler, Bolker, & Walker, 2014). The Laplace approximation of the log‐likelihood was used to estimate model parameters (Raudenbush, Yang, & Yosef, 2000). We included the fixed effects of treatment (i.e., pH of sperm‐EC chamber), fertilization design (standardized or unstandardized) and their interaction, and random effects of block and male ID (males nested within blocks; male IDs were coded uniquely to reflect this). The initial model was overdispersed (residual deviance = 346.12 on 98 *df*; dispersion factor = 3.53); to account for this, we added an observation‐level random effect to our final model (residual deviance = 21.13 on 97 *df*; dispersion factor = 0.22). The scaled residuals from the final model (calculated in the “DHARMa” package; Hartig, 2017) were uniformly distributed (Kolmogorov–Smirnov test; *D* = 0.062, *p* = .812). The significance of the fixed effects were initially assessed using Type III Wald chi‐Square tests in the “car” package (Fox & Weisberg, 2011); given the lack of significant interaction between the fixed effects (see Results), final tests of the main effects were Type II Wald chi‐Square (although the conclusions for the main effects did not differ across Type II and Type III tests). To test the significance of random effects, we removed each random effect in turn and compared the fit of the reduced models to the full model using likelihood ratio tests (likelihood ratio statistic *G* ^2^ = −2 × difference in log‐likelihoods, compared against χ^2^ distribution with 1 *df*). Aikaike information criteria with correction for finite sample sizes (AICc) were also calculated for full and reduced models. The full model, that is including all random effects, was used when testing the significance of fixed effects.

## 3 RESULTS

### 3.1 Seawater carbonate chemistry

Our manipulation of carbonate chemistry resulted in pH_T_ of 7.94 ± 0.01 (mean ± *SE*) in the ambient FSW and 7.55 ± 0.01 in the treated FSW (see Table 1 and Table S1 for corresponding Pco_2_ and DIC in each treatment), with a pH_T_ difference between FSW treatments of 0.39 ± 0.01 (mean ± *SE*) maintained across blocks (paired *t* test, *t*_5_ = 28.87, *p* < .001). Total alkalinity was not affected by the experimental manipulation (Table 1; paired *t* test, *t*_5_ = 0.91, *p* = .406).

**Table 1:** Carbonate chemistry parameters (mean ± standard error across experimental blocks) of each filtered seawater treatment

Treatment	pH_T_	A_T_ (µm/kg)	DIC (µm/kg)	Pco_2_ (µatm)
Ambient	7.94 ± 0.01	2,408 ± 16	2,183 ± 14	557 ± 20
Treated	7.55 ± 0.01	2,400 ± 24	2,339 ± 24	1,527 ± 27

Note

pH on the total scale (pH_T_) and total alkalinity (TA) were measured in the treatments for each block (*n* = 6 blocks), and dissolved inorganic carbon (DIC) and partial pressure of CO_2_ (Pco_2_) were calculated from pH_T_, TA, salinity, and temperature. Measured and calculated parameters for each individual seawater batch, along with propagated uncertainties in calculations, are provided in Table S1.

### 3.2 Sperm‐EC and fertilization trials

**Figure 2:**
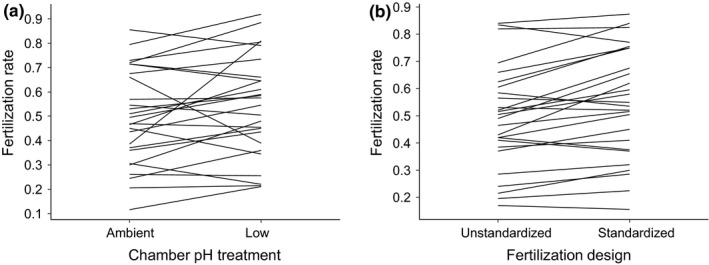
The fertilization rate (proportion fertilized out of 100 haphazardly sampled eggs) for each male (males represented by lines) following sperm‐EC trials across (a) seawater pH treatment in the sperm‐EC chamber (ambient pH = 8.0, low pH = 7.6) and (b) fertilization design (unstandardized or standardized pH in the fertilization mix; see Methods). Sample size *n* = 26 males

There was no detectable interaction between treatment and fertilization design (Wald $\chi_{1}^{2}$ = 0.39, *p* = .532), although both factors had significant main effects. The probability of fertilization was 6% higher in the low pH treatment compared with ambient pH (Wald $\chi_{1}^{2}$ = 7.11, *p* = .008; Figure 2a). Additionally, the probability of fertilization in the unstandardized fertilization pools was 7% lower than in the standardized pools (Wald $\chi_{1}^{2}$ = 8.31, *p* = .004; Figure 2b).

A comparison of model fits with and without each random effect revealed that there was significant variation in the probability of fertilization among males, but not among blocks (Table 2). This indicates that individual males had different mean fertilization rates, which could have been due to differences in sperm responses to ECs in the chambers and/or differences in ability to fertilize the eggs of standard females. A statistical test of the variation in response to treatments among males was beyond the scope of our study; however, there was some indication that the magnitude (and in some cases the direction) of difference between treatments might vary among individual males (Figure 2a).

**Table 2:** Results of log‐likelihood ratio tests comparing the fit of reduced models without each random effect (male and block) to the full model

Model	Log‐likelihood	AIC_c_	*G* ^2^	*p*
Full	−416.17	847.51		
(‐Male)	−449.73	912.33	67.12	<.001
(‐Block)	−416.49	845.85	0.64	.424

Note

Generalized linear mixed models were fit with proportion of fertilized eggs as the response variable, using a logit link function. The full model included fixed effects of chemotaxis treatment, fertilization design and their interaction, and random effects of block and male. Aikaike information criteria with correction for finite sample sizes (AICc) are provided for full and reduced models**.** The likelihood ratio statistic (*G* ^2^) for each random effect was calculated as −2 × difference in log‐likelihoods between the relevant reduced model and the full model and compared with a *χ* ^2^ distribution with 1 *df*.

## 4 DISCUSSION

Our results provide novel evidence that experimental adjustments in seawater pH affect sperm during prefertilization interaction with ECs, causing subsequent alterations to fertilization rates. Interestingly, fertilization rates were elevated under lower pH conditions. These patterns were consistent in both the unstandardized and standardized pH fertilization assays (although there were also overall higher fertilization rates in the standardized than unstandardized fertilization design, independent of the pH treatment effect). We can therefore attribute the pH treatment effect to the difference in pH of the sperm‐EC chambers, rather than any pH differences in fertilization mixes.

Our finding that fertilization rates were elevated under acidified conditions could indicate that (a) sperm responded more strongly to ECs under a low pH (e.g., more sperm accumulated at the source of the EC gradient, or capacitation and the acrosome reaction were stronger in response to ECs), and/or (b) sperm were better prepared for fertilization after swimming in low pH independent of their response to ECs. We consider the former possibility as a more likely explanation for the differential response of sperm across pH treatments, given the important role that ECs play in affecting the fertilization ability of sperm (Kekäläinen & Evans, 2016; Lymbery et al., 2017) and the likely sensitivity of sperm‐egg chemical signalling to seawater pH changes (Foo, Deaker, et al., 2018). Additionally, previous work on *M. galloprovincialis* revealed lower fertilization rates after sperm and eggs had been separately pre‐exposed to low pH environments (Eads et al., 2016), although the individual effects of pH on fertilization ability of eggs and sperm were not isolated in that study. Our current results, when combined with previous studies, indicate that effects of OA on reproductive success are likely to be complex and multifaceted and could act in different directions at different stages of sperm‐egg interactions.

The key advance of our study is that it isolates the effects of changes in seawater pH on fertilization to an early phase of the sperm‐egg interaction, rather than measuring sperm motility in isolation (i.e., in the absence of eggs or ECs) or using in vitro fertilization assays. Sperm‐egg chemical communication plays a key role in mediating natural fertilizations in a broad array of taxa (Eisenbach, 1999; Evans & Sherman, 2013; Miller, 1985). However, subtle effects of changing environmental conditions during sperm‐EC interactions are unlikely to be detected during standard in vitro fertilization assays, where sperm are mixed directly with eggs. Recent studies have suggested that communication via ECs could be adversely affected by OA. For example, in the sea urchins *Arabia lixula* and *Heliocidaris tuberculata*, OA reduces the size of the egg jelly coat, which contains sperm activating chemicals (Foo, Byrne, et al., 2018; Foo, Deaker, et al., 2018). In *H. tuberculata*, when sperm were mixed in a solution containing homogenized egg chemicals, sperm motility also differed across pH treatments, which could suggest that normal egg‐finding behavior is compromised under OA (Foo, Deaker, et al., 2018). In both *A. lixula* and *H. tuberculata*, therefore, the assumption is that a reduction in pH should reduce sperm success. By contrast, our study of *M. galloprovincialis* indicates that sperm fertilization success is enhanced after swimming in an EC gradient at low pH. This could reflect differences in OA effects among taxa (e.g., Foo, Deaker, et al., 2018 reported that OA effects on egg jelly coats were not consistent even across *Heliocidaris* sister species), or simply that the outcomes of sperm‐EC interactions are difficult to predict until explicitly tested. However, together these studies highlight the need to incorporate mechanistic knowledge of prefertilization sperm‐egg interactions into studies of OA effects.

The observed alteration of average sperm success under acidified conditions does not necessarily mean that populations impacted by OA will exhibit net positive increases in mean fitness. Instead, the fitness consequences of OA will depend on whether the observed effect disrupts the capacity of eggs to promote fertilizations by preferred sperm. In *M. galloprovincialis*, females use ECs to differentially regulate sperm movement and physiology (Evans et al., 2012; Kekäläinen & Evans, 2016; Oliver & Evans, 2014)—processes that ultimately favor sperm from genetically compatible males when multiple ejaculates compete for fertilization (as is likely in realistic mass spawning events) (Lymbery et al., 2017). This form of gamete‐mediated mate choice provides benefits to females in terms of enhanced offspring viability (Oliver & Evans, 2014). Therefore, increased overall fertilization capacity of sperm under acidified conditions could affect patterns of gamete‐mediated mate choice, with implications for offspring fitness. Even if the OA effect does not directly influence eggs or their ECs, there might be among‐male variation in sperm responses to ECs at low pH. While testing for among‐male variation in treatment effects was beyond the scope of this study, there are indications in our data of male‐specific responses to OA. Indeed, individual variation in OA effects is being increasingly reported in studies of sperm motility and in vitro fertilizations of other taxa (e.g., Schlegel et al., 2012; Schlegel, Havenhand, Obadia, & Williamson, 2014). Variable male responses to changes in ocean pH may further disrupt patterns of differential sperm‐EC interaction and therefore the ability of females to select sperm from compatible males. There is a clear need for future studies that determines whether the effects of OA on gamete interactions vary among males and male–female crosses (ideally under sperm‐competitive conditions) in order to better understand the fitness implications of changes in ocean pH for male and female fitness.

Ocean acidification may impact several stages of sperm‐EC communication, none of which is mutually exclusive. For example, the pH of seawater might (a) alter the capacity of eggs to control the amount or composition of ECs they release, (b) interact with EC profiles after they are released, or (c) affect the ability of sperm to recognize and respond to EC molecules. To tease apart these possibilities, we require a mechanistic understanding of sperm chemoattraction in *M. galloprovincialis*. In particular, we need to understand the chemical properties of ECs, ideally under different levels of acidification. To date, only a few EC molecules have been identified in broadcast spawning species (e.g., Böhmer et al., 2005; Riffell, Krug, & Zimmer, 2002; Ward, Brokaw, Garbers, & Vacquier, 1985; Yoshida, Murata, Inaba, & Morisawa, 2002) and we currently have no information in this regard for *M. galloprovincialis*. Furthermore, it would be interesting to examine the effect of OA on EC‐induced changes in sperm physiology, for example, pH_i_ changes and influx of calcium when mixed with ECs (e.g., see the techniques used in Kekäläinen & Evans, 2016; Kekäläinen, Larma, Linden, & Evans, 2015). Such studies would provide a detailed understanding of cellular and biochemical processes underlying the effects of OA on gamete interactions.

The difference between our two treatments represents upper predictions of near‐future change in mean seawater pH, consistent with much of the literature regarding the effects of OA on reproduction in marine species (Byrne, 2011). We note that future populations may have evolved in response to selection under acidified conditions and their gametes may act differently to those of current populations. However, for most species, it is unknown whether adaptation will match the rate of anthropogenic climatic change; this will depend on the amount of genetic variation in the relevant traits and the presence of genetic correlations with other fitness‐affecting traits (Hoffmann & Sgrò, 2011; Munday, Warner, Monro, Pandolfi, & Marshall, 2013; Pandolfi, Connolly, Marshall, & Cohen, 2011). Trade‐offs between resistance to climate change stressors and other fitness‐related traits, which are likely to limit responses to selection, have been reported in both marine and terrestrial organisms (e.g., Etterson & Shaw, 2001; Little, van Oppen, & Willis, 2004). A complete understanding of the consequences of OA for gamete interactions will require both (a) studies such as ours that measure effects of low pH on gametes from present populations and (b) estimates of genetic variances and covariances in relevant gamete traits.

In addition to the pH treatment effect, we found a main effect of fertilization design on fertilization rates, with higher overall fertilization rates in the standardized fertilization mix than in the unstandardized mix. This finding is difficult to interpret biologically; it is unlikely to be due to differences in pH, as the average pH of the fertilization mixes in the two designs should be equivalent (i.e., fertilization mixes in the standardized design should be midway between the ambient and treated conditions in the unstandardized design). One possibility is that the higher fertilization rates in the standardized mixes may be due to the higher volume of seawater and therefore lower gamete and embryo concentrations (although sperm:egg ratios remained the same). Previous studies have found negative relationships between embryo or larval density and survival in marine species (e.g., Johnson, 2008; Marshall & Evans, 2007), and it is possible that similar processes occur during very early development of fertilized eggs. Regardless of the causal mechanisms underlying this effect, it did not influence the relationship between OA and sperm success following EC trials.

In conclusion, we provide an investigation of how OA affects prefertilization sperm‐egg interactions and subsequent reproductive success. We show that sperm fertilization success after swimming in an EC gradient is altered under acidic conditions that are designed to simulate high‐CO_2_ environments. The direction of this effect highlights the need to incorporate a mechanistic understanding of sperm‐egg interactions into studies of OA. Future work should also focus on the implications of the observed effect for individual reproductive success of males and females, particularly with regards to the ability of females to mediate competition among multiple ejaculates. A comprehensive understanding of the complex patterns underlying fertilization success in marine systems will provide considerable power to predict the impacts of OA on reproduction and population persistence.

## AUTHOR CONTRIBUTIONS

R.A.L. and J.P.E. conceived of the study; R.A.L., J.P.E., and W.J.K. designed the experiment; R.A.L. and C.E.C. planned the manipulation and measurement of seawater carbonate chemistry; R.A.L. conducted the experiments, data collection, and statistical analyses; R.A.L. wrote the first draft of the manuscript, and all authors contributed to the final version. All authors gave final approval for publication.

## Data Availability

The data associated with this manuscript have been deposited in the Dryad Digital Repository (https://doi.org/10.5061/dryad.37pvmcvf2).
