# Supplementary material for: Ocean acidification during prefertilization chemical communication affects sperm success
Source: Ecol Evol. 2019 Oct 16;9(21):12302–10. doi: 10.1002/ece3.5720 (PMC6854328; doi:10.1002/ece3.5720)
Supplement: Supplementary file 1 [file ECE3-9-12302-s001.docx]

**SUPPLEMENTARY MATERIALS**

**Ocean acidification alters the strength of sperm chemoattraction**

Rowan A. Lymbery, W. Jason Kennington, Christopher E. Cornwall, Jonathan P. Evans

**Supplementary Table 1.** Carbonate chemistry parameters for each treatment of filtered seawater (control and ambient) in each experimental block (B1-6). pH and total alkalinity (A_T_) were measured in each sample, while dissolved inorganic carbon (DIC) and partial pressure of CO_2_ were calculated from the pH, A_T_, salinity and temperature of each sample. Propagated uncertainties for the calculations of DIC and pCO_2_ are shown in parentheses; these were estimated using the Gaussian method described in Orr, Epitalon, Dickson, & Gattuso (2018), implemented in the ‘seacarb’ package (Gattuso, Epitalon, Lavigne, & Orr, 2018) in R version 3.5.1 (R Core Team, 2018). We used conservative estimates for standard uncertainties of the measured parameters: pH = 0.02 (Orr et al. 2018 use 0.01 as an approximation of total standard uncertainty in potetiometric pH measurements) and A_T_ = 6 µm kg^-1^ (based on the difference between measured and expected values of certified reference material). Other parameters were set to the defaults described in the ‘seacarb’ package.

| Sample | pH | A_T_ (µm kg^-1^) | DIC (µm kg^-1^) | pCO_2_ (µatm) |
| --- | --- | --- | --- | --- |
| B1 Ambient | 7.98 | 2434.68 | 2195.97 (13.20) | 507.88 (29.15) |
| B1 Treated | 7.55 | 2453.06 | 2397.50 (9.56) | 1553.38 (81.77) |
| B2 Ambient | 7.96 | 2438.91 | 2206.74 (13.06) | 539.96 (30.89) |
| B2 Treated | 7.57 | 2432.99 | 2369.01 (9.61) | 1470.29 (77.68) |
| B3 Ambient | 7.97 | 2368.24 | 2142.25 (12.75) | 505.05 (28.88) |
| B3 Treated | 7.54 | 2369.44 | 2318.93 (9.36) | 1546.96 (81.34) |
| B4 Ambient | 7.93 | 2426.01 | 2196.47 (13.00) | 573.33 (32.79) |
| B4 Treated | 7.54 | 2425.10 | 2361.47 (9.66) | 1567.67 (82.87) |
| B5 Ambient | 7.90 | 2434.33 | 2218.69 (12.68) | 630.74 (35.80) |
| B5 Treated | 7.54 | 2421.75 | 2358.08 (9.67) | 1598.93 (84.54) |
| B6 Ambient | 7.91 | 2351.59 | 2137.02 (12.49) | 586.15 (33.36) |
| B6 Treated | 7.56 | 2295.80 | 2228.51 (9.45) | 1426.56 (75.63) |

**Supplementary References**

Gattuso, J.-P., Epitalon, J.-M., Lavigne, H., & Orr, J. (2018). seacarb: Seawater Carbonate Chemistry. R package version 3.2.8. http://CRAN.R-project.org/package=seacarb.

Orr, J. C., Epitalon, J.-M., Dickson, A. G., & Gattuso, J.-P. (2018). Routine uncertainty propagation for the marine carbon dioxide system. *Marine Chemistry*, *207*, 84–107.

R Core Team. (2018). R: A language and environment for statistical computing. Vienna, Austria. Available online at http://www.R-project.org: R Foundation for Statistical Computing. Retrieved from http://www.r-project.org/
